# Holding a stigmatizing attitude at the start of the COVID‐19 outbreak: A cross‐sectional survey

**DOI:** 10.1111/bjhp.12564

**Published:** 2021-10-04

**Authors:** Louise E. Smith, Henry W. W. Potts, Richard Amlȏt, Nicola T. Fear, Susan Michie, G. James Rubin

**Affiliations:** ^1^ King’s College London Institute of Psychiatry, Psychology and Neuroscience UK; ^2^ NIHR Health Protection Research Unit in Emergency Preparedness and Response UK; ^3^ University College London Institute of Health Informatics UK; ^4^ Emergency Response Department Science and Technology Public Health England Behavioural Science Team UK; ^5^ Academic Department of Military Mental Health King’s Centre for Military Health Research UK; ^6^ Centre for Behaviour Change University College London UK

**Keywords:** COVID‐19, discrimination, infectious disease outbreak, stigma, worry

## Abstract

**Objectives:**

To identify the prevalence of a stigmatizing attitude towards people of Chinese origin at the start of the COVID‐19 outbreak in the UK population and investigate factors associated with holding the stigmatizing attitude.

**Design:**

Online cross‐sectional survey conducted 10–13 February 2020 (*n* = 2006, people aged 16 years or over and living in the UK).

**Methods:**

We asked participants to what extent they agreed it was best to avoid areas heavily populated by Chinese people because of the COVID‐19 outbreak. Survey materials also asked about: worry, perceived risk, knowledge, information receipt, perception of government response to COVID‐19, and personal characteristics. We ran binary logistic regressions to investigate associations between holding a stigmatizing attitude, personal characteristics, and psychological and contextual factors.

**Results:**

26.1% people (95% CI 24.2–28.0%, *n* = 524/2006) agreed it was best to avoid areas heavily populated by Chinese people. Holding a stigmatizing attitude was associated with greater worry about COVID‐19, greater perceived risk of COVID‐19, and poorer knowledge about COVID‐19.

**Conclusions:**

At the start of the COVID‐19 pandemic, a large percentage of the UK public endorsed avoiding areas in the UK heavily populated by people of Chinese origin. This attitude was associated with greater worry about, and perceived risk of, the COVID‐19 outbreak as well as poorer knowledge about COVID‐19. At the start of future novel infectious disease outbreaks, proactive communications from official sources should provide context and facts to reduce uncertainty and challenge stigmatizing attitudes, to minimize harms to affected communities.

**Statement of contribution**

***What is already known on this subject***

- Stigmatizing attitudes are common at the start of novel infectious disease outbreaks.
- Groups perceived as being responsible for the origin and spread of infection are often blamed.
- At the start of the COVID‐19 outbreak, people of Chinese origin and appearance were stigmatized.

***What this study adds***

- At the start of the COVID‐19 outbreak, 26% of the UK public endorsed a stigmatizing attitude.
- Holding a stigmatizing attitude was associated with greater worry about, and greater perceived risk of, COVID‐19.
- Holding a stigmatizing attitude was also associated with poorer knowledge about COVID‐19.

## Background

Stigma has been defined as occurring when people distinguish and label differences, link these differences to stereotypes, and separate themselves from the ‘other’ group, leading to discrimination and loss of status (Link & Phelan, 2001). Stigmatization at the start of infectious disease outbreaks is common and fuelled by fear of the unknown and the association of unknowns with ‘others’ (International Federation of Red Cross & Red Crescent Societies, UNICEF, World Health Organization, 2020; The Independent Scientific Advisory Group for Emergencies (SAGE), 2021). For example, outbreaks of novel infectious diseases are often characterized by a pattern of distancing the disease from oneself or in‐groups, blaming groups perceived as responsible for the origin and spread of infection (often marginalized groups or those in power, such as the government), and stigmatization of those who have contracted the illness or who are thought to have exacerbated the spread (Des Jarlais, Galea, Tracy, Tross, & Vlahov, 2006; Joffe, 2011; Person et al., 2004). As all outbreaks of novel infectious diseases start in a similar manner – as an unknown entity – it is important to understand factors that may contribute to stigmatizing attitudes, to inform policy and communications which aim to minimize the impact of stigma at the start of future outbreaks.

The COVID‐19 pandemic emerged from Wuhan, China. On 23 January 2020, the Chinese government imposed a lockdown on Wuhan and other cities in Hubei province (Kuo, 2020). By 11 February 2020, there had been 42,708 confirmed COVID‐19 cases and 1017 deaths in China, and the virus had been detected in 24 other countries (total 393 cases and 1 death) (WHO Director‐General, 2020). Between 29 January and 27 February 2020, there were over 50 repatriation flights from Wuhan (Thompson et al., 2020), including two to the UK that received widespread media attention (Justin & Ratcliffe, 2020). On 1 April 2020, the UN Secretary General dubbed the COVID‐19 pandemic as the ‘most challenging crisis’ for the world since World War II (News Wires, 2021).

At the start of the COVID‐19 outbreak, influential figures around the world referred to the virus based on its place of origin (e.g., ‘Wuhan coronavirus’, ‘China virus’) (Viladrich, 2021), increasing stigmatization and discrimination (Hswen et al., 2020). Internationally, there was an increase in racism towards people of Chinese or East Asian descent (Schumann & Moore, 2021; Villa et al., 2020). The latest census (conducted in 2011) found that people identifying as Chinese made up 0.7% of the population of England and Wales, with 31.6% of people of Chinese origin living in London (GOV.UK, 2020a, 2020b). In the UK, reported rates of hate crimes towards people of Chinese origin or appearance in 2020 was two to three times higher than that in the previous 2 years (Mayor of London, 2020; Metropolitan Police, 2020).

While factors associated with perceiving stigma have been well‐researched, there is less research investigating factors associated with endorsing stigmatizing attitudes. Research suggests that holding stigmatizing attitudes towards medical conditions (e.g., HIV/AIDS) is associated with sociodemographic characteristics, such as lower education, and psychological factors, such as poorer knowledge about the condition (Li et al., 2017). Beliefs that someone has personal control, responsibility, and blame over having contracted an illness have also been found to explain endorsement of stigmatizing attitudes towards infectious diseases (Mak et al., 2006).

In this study, we investigated the prevalence of holding a stigmatizing attitude towards the Chinese community in the UK at the start of the COVID‐19 outbreak. We investigated whether holding a stigmatizing attitude was associated with personal characteristics and psychological and contextual factors.

## Method

### Design

Online cross‐sectional survey conducted by BMG research on behalf of the English Department of Health and Social Care (data collected 10–13 February 2020). We analysed these data as part of the COVID‐19 Rapid Survey of Adherence to Interventions and Responses (CORSAIR) study (Smith, Potts, Amlôt, et al., 2021). In the UK, the first two cases of COVID‐19 were declared on 31 January 2020. At the time of data collection, there had been a total of nine cases detected. Of these cases, four were transmitted in East Asia and five were contacts of a confirmed UK case where the virus was transmitted in France. No onward transmission had been detected within the country.

### Participants

Participants were recruited from Respondi, a specialist research panel provider (*n* = 50,000), and were eligible for the study if they were aged 16 years or over and lived in the UK. Quotas based on age and gender (combined) and Government Office Region reflected targets based on the Office for National Statistics. For this survey, participants were reimbursed in points (equivalent to approximately 25p) which could be redeemed in cash, gift vouchers, or charitable donations. For this analysis, we had a final sample of 2006. Because quick turn‐around for data collection is essential during a rapidly evolving crisis, the survey used standard opinion polling methods using non‐probability sampling, an approach common within market research, political polling, and social science.

### Study materials

#### Outcome measure

Participants were asked to what extent they agreed that ‘because of the coronavirus outbreak, it is best to avoid areas in the UK that are heavily populated by Chinese people’ on a five‐point ‘strongly disagree’ to ‘strongly agree’ scale, which was recoded to create a binary outcome variable (‘strongly agree’ and ‘agree’ versus ‘strongly disagree’, ‘disagree’ and ‘neither agree nor disagree’). Participants could also answer ‘don’t know’; these answers were recoded as missing for our binary outcome variable.

#### Personal characteristics

Participants were asked to state: their age; gender; whether they had dependent children; whether they themselves or another household member had a chronic illness; their employment status; whether they themselves, a family member, or friend worked for the National Health Service (NHS); their highest level of education; and their ethnicity. Index of multiple deprivation was derived from participants’ postcode (Ministry of Housing Communities & Local Government, 2019).

#### Psychological and contextual factors

##### Worry

Worry about COVID‐19 was measured by a single item asking participants ‘overall, how worried are you about coronavirus?’. Responses were on a five‐point scale from ‘not at all worried’ to ‘extremely worried’. We recoded worry about coronavirus as a binary variable, grouping together ‘very’ or ‘extremely worried’ versus ‘not at all’, ‘not very’, or ‘somewhat worried’.

##### Perceived risk

Perceived risk about COVID‐19 was measured by asking participants ‘to what extent [they] thought coronavirus [posed] a risk’ to themselves and people in the UK on a five‐point scale from ‘no risk at all’ to ‘major risk’. Participants were also asked to what extent ‘coronavirus would be a serious illness for me’ on a five‐point scale (‘strongly disagree’ to ‘strongly agree’).

##### Knowledge

To measure knowledge about COVID‐19, participants were asked to what extent they agreed or disagreed with seven items relating to misinformation that was being spread at the time of data collection (five‐point scale: ‘strongly disagree’ to ‘strongly agree’). These were as follows:

- I could catch coronavirus from animals [false]
- I could catch coronavirus from packages or products ordered from China [false]
- I could catch coronavirus from someone else who has it, even if they do not have any symptoms yet [true]
- It is likely that I have some natural immunity to coronavirus [false]
- There is a vaccine available to protect against coronavirus [false]
- Antibiotics are an effective treatment for coronavirus [false]
- It is currently unsafe to come into contact with someone who has been to Wuhan in China in the past 14 days, regardless of whether they seem ill or well [true].

We judged responses as ‘true’ or ‘false’ based on information provided at the time by the UK Government. We scored knowledge items from +2 (strong agreement with a correct answer) to −2 (strong disagreement with a correct answer) and coded ‘don’t know’ as 0. We summed the items to give a total knowledge score, rescaled to give a score of 1 to 29, with higher scores indicating higher knowledge.

##### Information

Participants were asked how much they had ‘seen or heard about coronavirus in the past seven days’ with possible responses being ‘I have not seen or heard anything’, ‘I have seen or heard a little’, ‘I have seen or heard a fair amount’, and ‘I have seen or heard a lot’. On 2 February 2020, a public information campaign was launched by the English Department of Health and Social Care called ‘Catch it, Bin it, Kill it’ (Department of Health & Social Care, 2019), based on a similar campaign of the same name developed in the 2009/10 influenza H1N1 pandemic. Participants were asked if they had seen or heard ‘advice on how to protect yourself and others from coronavirus’ and ‘recommendations to “Catch it, Bin it, Kill it”’ in the last seven days. Possible answers were ‘yes, I have seen or heard this’ and ‘no, I haven’t seen or heard this’.

Participants were asked to identify the three sources that they had ‘received most of [their] information about coronavirus from in the past seven days’ from a list of sixteen possible sources. These included official sources such as NHS111 (a free‐to‐call single non‐emergency number medical helpline operating in most of the UK), the NHS website, and GOV.UK (the UK government website); mainstream media, such as television news, newspapers (print and online), and radio; and unofficial sources, for example, social media sites, search engines, and friends and relatives. We created separate binary variables to indicate whether participants had received most of their information from official sources, the mainstream media, or unofficial sources. For each information source, participants were said to have used this source if they indicated it as one of their top three sources of information.

Government response: Participants were asked to state to what extent they agreed or disagreed that: ‘the Government [was] putting the right measures in place to protect the British public from coronavirus’; they felt they were ‘getting the information [they needed] from the Government and other public authorities on coronavirus’; and they felt they knew what they needed to do ‘to limit [their] risk of contracting coronavirus’. Participants answered on a five‐point Likert scale (‘strongly disagree’ to ‘strongly agree’). We summed scores on these three items to give a total score (range 3–15, Cronbach’s α = .74). Lower scores indicated lower satisfaction with the Government.

To assess perceived credibility of information from the Government, participants completed an adapted form of the Meyer Credibility Index (Meyer, 1988). Participants were asked to state on a five‐point scale Likert scale (‘strongly disagree’ to ‘strongly agree’) whether information from the Government about coronavirus could be trusted, was accurate, told the whole story, and was biased or one‐sided. We summed scores on the four items of the Meyer Credibility Index items (range 4–20, Cronbach’s α = .76). Lower scores indicated poorer credibility.

### Ethics

This work was conducted as a service evaluation of the Department of Health and Social Care’s public communications campaign and, following advice from the University Research Ethics Sub‐committee, was exempt from ethical approval.

### Patient and public involvement

Due to the rapid nature of data collection, patients and public were not involved in the design, analysis, or interpretation of results. The survey questions were based on materials developed in 2014 in preparation for a future influenza pandemic by our team (Simpson et al., 2019). These items were refined in 2014 in three rounds of qualitative interviews (*n* = 78) and had their test–retest reliability checked in two telephone surveys (*n* = 621; Rubin et al., 2014).

### Power

A target sample size of 2,000 was used for each wave, allowing a 95% confidence interval of, at most, plus or minus 2.2% for the prevalence estimate for each survey item.

### Analysis

We used binary logistic regressions to calculate univariable associations between a stigmatizing attitude and personal characteristics, worry, perceived risk, knowledge, information, and perception of government response. We used a second set of logistic regressions adjusting for all personal characteristics.

We hypothesized that worry would be associated with uptake of preventive behaviours and stigmatization (Rubin, Potts, & Michie, 2010; Smith, Potts, Amlot, et al., 2021). Therefore, we ran *post hoc* logistic regression analyses adjusting for worry about coronavirus as well as personal characteristics.

We recoded answers of ‘don’t know’ as missing data.

The survey method used quota sampling with weightings. In practice, the weights did not substantially affect rates of holding a stigmatizing attitude. Our analyses report unweighted statistics.

## Results

26.1% (95% CI 24.2–28.0%, *n* = 524/2006) agreed that it was best to avoid areas in the UK that were heavily populated by Chinese people; 64.3% (95% CI 62.2–66.4%, *n* = 1290/2006) did not agree; and 9.6% did not know (95% CI 8.3–10.9%, *n* = 92/2006; see Table 1 for breakdown).

**Table 1:** Percentage of people who agreed or disagreed that because of the COVID‐19 outbreak, it was best to avoid areas in the UK that were heavily populated by Chinese people (total *n* = 2006)

	*N*	% (95% CIs)
Strongly agree	208	10.4 (9.0–11.7)
Agree	316	15.8 (14.2–17.3)
Neither agree nor disagree	396	19.7 (18.0–21.5)
Disagree	524	26.1 (24.2–28.0)
Strongly disagree	370	18.4 (16.7–20.1)
Don’t know	192	9.6 (8.3–10.9)

Results of univariable and multivariable analyses are reported in Tables 2 and 3. Holding a stigmatizing attitude was associated with: greater worry about COVID‐19; greater perceived risk from COVID‐19 (to oneself and people in the UK); greater perceived severity of COVID‐19; poorer knowledge about COVID‐19; not having seen or heard information from mainstream media; having seen or heard information from official sources; greater satisfaction with the UK Government response; having a chronic illness oneself; having a dependent child in the household; being employed; lower education; and living in a more deprived area. Younger age was associated with holding a stigmatizing attitude in a non‐linear manner, with stigmatizing attitude declining with increasing age and then flattening.

**Table 2:** Associations between personal characteristics and holding a stigmatizing attitude

Participant characteristics	Level	Because of the coronavirus outbreak, it is best to avoid areas in the UK that are heavily populated by Chinese people			
		Neither agree not disagree/disagree/strongly disagree *n* = 1,290, *n* (%)	Agree/strongly agree *n* = 524, n (%)	Odds ratio (95% CI) for holding a stigmatizing attitude	Adjusted odds ratio (95% CI) for holding a stigmatizing attitude
Gender	Male	624 (68.6)	285 (31.4)	Reference	Reference
	Female	657 (73.4)	238 (26.6)	0.79 (0.65–0.97)*	0.83 (0.67–1.02)
Age	*N, M, SD*	*N* = 1,290, *M* = 48.9, *SD* = 18.2	*N* = 524, *M* = 45.6, *SD* = 19.2	0.99 (0.98–1.00)*	0.93 (0.90–0.96)*
Age – quadratic (age‐mean)^2^	–	–	–	–	5.61 (2.42–13.02)*
Dependent children	No	932 (73.6)	334 (26.4)	Reference	Reference
	Yes	358 (65.3)	190 (34.7)	1.48 (1.19–1.84)*	1.48 (1.15–1.91)*
Chronic illness – self	None	903 (72.8)	338 (27.2)	Reference	Reference
	Present	371 (67.7)	177 (32.3)	1.27 (1.02–1.59)*	1.51 (1.19–1.93)*
Chronic illness – other household member	None	1,093 (71.7)	432 (28.3)	Reference	Reference
	Present	181 (68.6)	83 (31.4)	1.16 (0.87–1.54)	1.12 (0.83–1.52)
Employment status	Not working	591 (73.7)	221 (26.3)	Reference	Reference
	Working	687 (69.0)	308 (31.0)	1.26 (1.02–1.54)*	1.38 (1.06–1.81)*
Work for NHS – self	No	1,204 (72.0)	468 (28.0)	Reference	Reference
	Yes	75 (62.5)	45 (37.5)	1.54 (1.05–2.27)*	1.16 (0.77–1.77)
Work for NHS – members of my family	No	1,106 (70.6)	461 (29.4)	Reference	Reference
	Yes	173 (76.9)	52 (23.1)	0.72 (0.52–1.00)*	0.72 (0.51–1.01)
Work for NHS – friends	No	1,143 (70.9)	469 (29.1)	Reference	Reference
	Yes	126 (75.6)	44 (24.4)	0.79 (0.55–1.13)	0.75 (0.51–1.09)
Highest educational or professional qualification	GCSE/vocational/A‐level/No formal qualifications	832 (69.5)	365 (30.5)	Reference	Reference
	Degree or higher (Bachelors, Masters, PhD)	458 (74.2)	159 (25.8)	0.79 (0.64–0.98)*	0.75 (0.59–0.95)*
Index of multiple deprivation	1st quartile (least deprived)	309 (75.9)	98 (24.1)	Reference	Reference
	2nd quartile	327 (72.8)	122 (27.2)	1.18 (0.86–1.60)	1.05 (0.76–1.44)
	3rd quartile	330 (69.0)	148 (31.0)	1.41 (1.05–1.91)*	1.31 (0.96–1.79)
	4th quartile (most deprived)	324 (67.5)	156 (32.5)	1.52 (1.13–2.04)*	1.36 (1.00–1.87)*
Ethnicity	White	1,197 (71.8)	469 (28.2)	Reference	Reference
	Minoritized ethnic groups	82 (60.7)	53 (39.3)	1.65 (1.15–2.37)*	1.45 (0.97–2.15)

* *p* ≤ .05.

**Table 3:** Associations between psychological and contextual factors and holding a stigmatizing attitude

	Participant characteristics	Level	Because of the coronavirus outbreak, it is best to avoid areas in the UK that are heavily populated by Chinese people			
			Neither agree not disagree/disagree/strongly disagree *n* = 1,290, *n* (%)	Agree/strongly agree *n* = 524, *n* (%)	Odds ratio (95% CI) for holding a stigmatizing attitude	Adjusted odds ratio (95% CI) for holding a stigmatizing attitude
Worry	Worry	Not at all/not very/somewhat worried	1,101 (77.7)	316 (22.3)	Reference	Reference
		Very/extremely worried	181 (46.8)	206 (53.2)	3.97 (3.13–5.02)*	3.69 (2.86–4.75)*
Perceived risk	To oneself	5‐point Likert‐type (1=no risk at all, 5=major risk)	*N* = 1,268, *M* = 2.3, *SD* = 0.9	*N* = 514, *M* = 2.8, *SD* = 1.2	1.65 (1.49–1.83)*	1.56 (1.40–1.74)*
	To people in the UK	5‐point Likert‐type (1=no risk at all, 5=major risk)	*N* = 1,277, *M* = 2.8, *SD* = 0.9	*N* = 518, *M* = 3.4, *SD* = 1.1	1.94 (1.74–2.17)*	1.86 (1.66–2.09)*
	Severity of coronavirus (self)	5‐point Likert (1=strongly disagree, 5=strongly agree)	*N* = 1,205, *M* = 2.6, *SD* = 1.1	*N* = 492, *M* = 4.2, *SD* = 0.9	1.72 (1.54–1.93)*	1.75 (1.55–1.98)*
Knowledge	Knowledge	Range 6–29	*N* = 1,290, *M* = 20.1, *SD* = 3.4	*N* = 524, *M* = 18.0, *SD* = 4.6	0.86 (0.84–0.89)*	0.87 (0.84–0.89)*
Information	Amount heard	4‐point Likert‐type (1=have not seen or heard anything, 4=seen or heard a lot)	*N* = 1,286, *M* = 3.3, *SD* = 0.7	*N* = 523, *M* = 3.4, *SD* = 0.7	1.04 (0.90–1.20)	1.01 (0.87–1.18)
	Information source – official sources	No	1,024 (72.7)	384 (27.3)	Reference	Reference
		Yes	266 (65.5)	140 (34.5)	1.40 (1.11–1.78)*	1.33 (1.03–1.71)*
	Information source – mainstream media	No	119 (61.3)	75 (38.7)	Reference	Reference
		Yes	1,171 (72.3)	449 (27.7)	0.61 (0.45–0.83)*	0.59 (0.42–0.83)*
	Information source – unofficial sources	No	828 (71.7)	327 (28.3)	Reference	Reference
		Yes	462 (70.1)	197 (29.9)	1.08 (0.87–1.33)	0.87 (0.68–1.11)
	Advice on protection	No	454 (71.2)	184 (28.8)	Reference	Reference
		Yes	836 (71.1)	340 (28.9)	1.00 (0.81–1.24)	1.00 (0.80–1.25)
	Recommendations to ‘Catch it, Bin it, Kill it’	No	568 (71.4)	227 (28.6)	Reference	Reference
		Yes	722 (70.9)	297 (29.1)	1.03 (0.84–1.26)	1.06 (0.85–1.31)
Government response	Satisfaction with government response	Range 3 (lowest) to 15 (highest)	*N* = 1,132, *M* = 10.7, *SD* = 2.4	*N* = 466, *M* = 11.0, *SD* = 2.5	1.05 (1.00–1.10)*	1.08 (1.03–1.13)*
	Credibility of government	Range 4 (lowest) to 20 (highest)	*N* = 1,000, *M* = 13.3, *SD* = 3.1	*N* = 419, *M* = 13.3, *SD* = 3.1	1.01 (0.97–1.04)	1.03 (0.99–1.08)

* *p* ≤ .05.

In post hoc analyses which adjusted for worry and personal characteristics, the following factors were no longer associated with holding a stigmatizing attitude: employment status, index of multiple deprivation, and having seen or heard information from official sources. Greater credibility of information from the Government about COVID‐19 was additionally associated.

## Discussion

We found that approximately one‐quarter of the UK population held a stigmatizing attitude towards people from Chinese communities at the start of the COVID‐19 outbreak. Other UK surveys conducted at a similar time found that 14% reported avoiding contact with people of Chinese origin or appearance (Ipsos MORI, 2020) and that 30% thought it would be ‘prudent’ to not eat at Chinese restaurants ‘to reduce the risk of getting infected with coronavirus’ (Geldsetzer, 2020). This can lead to economic harm (Rubin et al., 2020; Sybilla & Cavataro, 2020) and may be associated with other trends such as an increase in hostility, including discrimination and racially aggravated assault (Mayor of London, 2020; Metropolitan Police, 2020; Yeh, 2020). Studies in other countries have also shown evidence for blaming of the Chinese community at the start of the COVID‐19 outbreak (Idoiaga Mondragon, Berasategi Sancho, Ozamiz‐Etxebarria, & Alonso, 2021). Over 18 months later, these stigmatizing attitudes are still evident in the UK population, with 40% opposing all tourists from China (regardless of vaccination status), compared to 20% and 28% opposition for tourists from Denmark and the USA, respectively (data collected 8 to 9 July 2021) (YouGov, 2021).

Holding a stigmatizing attitude was associated with greater perceived worry about COVID‐19 and higher perceptions of the risk and severity of COVID‐19. It may be that people who were more worried and perceived a greater risk from COVID‐19 thought they would be protect themselves from contracting the infection by avoiding areas frequented by Chinese populations in the UK. This highlights the need for official communications that provide clear advice on behaviours that prevent the spread of illness, such as good respiratory and hand hygiene and physical distancing (Ahmed, Zviedrite, & Uzicanin, 2018; Jefferson et al., 2020).

In line with research investigating outbreaks of other infectious diseases, we found an association between poorer knowledge about COVID‐19 and being more likely to hold a stigmatizing attitude (Li et al., 2017). At the time of data collection, there were many uncertainties surrounding COVID‐19. The majority of confirmed COVID‐19 cases and deaths had occurred in China (WHO Director‐General, 2020), and the worldwide news media were reporting on the strict quarantine measures that had been put in place in Wuhan and other cities in Hubei Province (Kuo, 2020). Our results suggest that at the start of an emerging infectious disease outbreak, there is a need for proactive official communications which provide contextual and factual information to reduce uncertainty, and which challenges emerging stigmatizing attitudes. As recommended by the WHO (World Health Organization, 2021), neutral scientific language to describe a pathogen, rather than deriving a name from its country of origin (e.g., ‘Wuhan coronavirus’, ‘China virus’), may help reduce negative sentiments towards that group (Hswen et al., 2020; Viladrich, 2021).

In our data, there seemed to be a trend between holding a stigmatizing attitude and receiving information from official sources. This association was no longer apparent when controlling for worry, suggesting that higher worry may have driven both stigma and information seeking. We found that those who had not seen or heard information from the mainstream media were more likely to hold a stigmatizing attitude. In the UK, most people use mainstream media for their news (77% television, 47% newspapers, 43% radio) (Jigsaw Research, 2020), and the minority who do not may be more likely to already hold stigmatizing attitudes or be more likely to encounter them in the non‐mainstream media sources that they turn to for news (Allington, Duffy, Wessely, Dhavan, & Rubin, 2020). We found an association between holding a stigmatizing attitude and greater satisfaction with the UK Government response; greater perceived credibility of the UK Government was associated with holding a stigmatizing attitude when adjusting for worry. We are not sure why this may be and tentatively speculate that these associations may be confounded by political beliefs (Duffy, Hewlett, Hesketh, Benson, & Wager, 2021). Our results differ from a study investigating attitudes towards HIV/AIDS, tuberculosis, and SARS in Hong Kong, which found that holding more stigmatizing attitudes was associated with less favourable attitudes towards Government policies related to the disease (Mak et al., 2006).

Holding a stigmatizing attitude was also associated with personal characteristics such as having a dependent child, lower education, having a chronic illness oneself, and younger age. With the exception of education, these factors were all associated with greater worry about COVID‐19 at the start of the outbreak (Smith, Potts, Amlot, et al., 2021). However, associations remained significant even after adjusting for worry. These groups may be more dependent on public services, with people with dependent children sending them to school or childcare and those with a chronic health condition using the healthcare system more, and may be more likely to pick up infection (through their children) or more likely to suffer severe illness (those with a chronic illness). Therefore, these groups may have been more likely to avoid people they perceived as being a source of infection. There is limited research investigating personal characteristics associated with holding a stigmatizing attitude, but one other study has also found that people with lower education are more likely to hold stigmatizing attitudes towards people living with an infectious disease (Li et al., 2017).

Stigmatizing attitudes due to infectious disease outbreaks persist over time, continuing on after the outbreak has been controlled (Viladrich, 2021). For example, one study found that people who had contracted SARS in Hong Kong in 2003 were still experiencing discrimination up to three years later (Siu, 2008). Therefore, it should not be assumed that the passage of time will resolve stigmatization and discrimination. This is concerning as experiencing racial discrimination is associated with greater psychological distress and poorer life satisfaction (Hackett, Ronaldson, Bhui, Steptoe, & Jackson, 2020). As at the start of other emerging infectious disease outbreaks, a stigmatizing attitude towards a minoritized ethnic group was prevalent in the UK at the start of the COVID‐19 outbreak. As the pandemic has progressed, stigmatizing attitudes may have shifted to other minoritized populations perceived as spreading the illness, people who have had infection confirmed, and those who show symptoms of infection (e.g., coughing in public) (The Independent Scientific Advisory Group for Emergencies (SAGE), 2021). This is worrying in the context of an emerging infectious disease outbreak, as fear of social stigma for catching infectious diseases can stop people seeking treatment (Williams, Gonzalez‐Medina, & Le, 2011). Stigmatizing attitudes towards people who are not vaccinated may also develop as the pandemic progresses.

Several limitations should be considered for this study. First, we used self‐report measures. Social desirability may have minimized reporting of the stigmatizing attitude. Second, while we did not directly ask whether participants held a stigmatizing attitude, due to the likely influence of social desirability on such an item, we asked participants whether they would ‘avoid areas heavily populated by Chinese people’. Economic secondary stressors often have a large and lasting influence following incidents (e.g., decreases in tourism and trade in the pork industry in Mexico following the emergence of H1N1 influenza (Rassy & Smith, 2013) and decreases in tourism following the Novichok poison incident in Salisbury (Clarke & Weir, 2020)), making this an outcome of particular interest. Third, while the use of an online market research panel is helpful in ensuring data are collected quickly, there are limitations to this approach. People who actively sign up for such panels may not be representative of the general public in terms of, for example, the amount of time they spend online and hence the likelihood of them encountering online public health campaigns. Quota samples aim to decrease response bias by filling pre‐determined targets so that the distribution of pre‐specified participant characteristics is representative of the wider population. As such, participants that belong to a quota that has already been met are prevented from completing the survey and response rate is not a useful indicator of response bias. Fourth, the cross‐sectional nature of the data makes it impossible to be certain about the directions of causality in the associations we have reported. Fifth, given the large number of statistical tests we conducted, type 1 errors may be apparent in our data and caution is particularly required for associations where the confidence intervals approach one. Sixth, location and proximity to areas heavily populated by the Chinese population may have influenced stigmatizing attitudes.

At the start of future infectious disease outbreaks, we need to expect, measure, and address the fact that groups perceived as responsible for the origin and spread of infection will experience stigmatization and discrimination. We found that at the start of the COVID‐19 outbreak in the UK, over one‐quarter of people held a stigmatizing attitude, namely thinking it was best to avoid areas heavily populated by Chinese people. Holding a stigmatizing attitude was associated with poorer knowledge, greater worry about COVID‐19, and greater perceived risk of COVID‐19. This suggests a need for proactive communications outlining contextual and factual information about disease transmission, evidence‐based information about behaviours which prevent the spread of infection, and challenging stigmatizing attitudes.

## Funding information

This work was funded by the National Institute for Health Research (NIHR) Health Services and Delivery Research programme. LS, RA, and GJR are supported by the National Institute for Health Research Health Protection Research Unit (NIHR HPRU) in Emergency Preparedness and Response, a partnership between Public Health England, King’s College London, and the University of East Anglia. RA is also supported by the NIHR HPRU in Behavioural Science and Evaluation, a partnership between Public Health England and the University of Bristol. HWWP receives funding from Public Health England and has from NHS England. NTF is part funded by a grant from the UK Ministry of Defence. The views expressed are those of the authors and not necessarily those of the NIHR, Public Health England, the Department of Health and Social Care, or the Ministry of Defence. Surveys were commissioned and funded by Department of Health and Social Care (DHSC), with the authors providing advice on the question design and selection. DHSC had no role in analysis, decision to publish, or preparation of the manuscript. Preliminary results were made available to DHSC and the UK’s Scientific Advisory Group for Emergencies.

## Conflict of interest

All authors had financial support from NIHR for the submitted work. RA is an employee of Public Health England; HWWP receives additional salary support from Public Health England and NHS England; HWWP receives consultancy fees to his employer from Ipsos MORI, and has a PhD student who works at and has fees paid by Astra Zeneca; and NTF is a participant of an independent group advising NHS Digital on the release of patient data. All authors are participants of the UK’s Scientific Advisory Group for Emergencies or its subgroups. There are no other financial relationships with any organizations that might have an interest in the submitted work in the previous three years and no other relationships or activities that could appear to have influenced the submitted work.

## Author Contribution

**Louise E Smith:** Conceptualization (equal); Data curation (equal); Formal analysis (equal); Methodology (equal); Writing – original draft (equal). **Henry WW Potts:** Conceptualization (equal); Funding acquisition (equal); Methodology (equal); Writing – review & editing (equal). **Richard Amlôt:** Conceptualization (equal); Funding acquisition (equal); Methodology (equal); Writing – review & editing (equal). **Nicola T Fear:** Conceptualization (equal); Funding acquisition (equal); Methodology (equal); Writing – review & editing (equal). **Susan Michie:** Conceptualization (equal); Funding acquisition (equal); Methodology (equal); Writing – review & editing (equal). **G James Rubin:** Conceptualization (equal); Funding acquisition (equal); Methodology (equal); Writing – review & editing (equal).

## Data Availability

The data are owned by the UK’s Department of Health and Social Care, so no additional data are available from the authors.
